# Jejunal Diverticular Bleeding on Long-Term Aspirin and Short-Term Corticosteroid Therapy

**DOI:** 10.1155/crgm/8875482

**Published:** 2024-11-14

**Authors:** Nikolaos Angelopoulos, William Beattie, Sern Wei Yeoh

**Affiliations:** ^1^Department of Anaesthesia and Intensive Care, Western Health, Melbourne, Victoria, Australia; ^2^Department of Gastroenterology, Western Health, Melbourne, Victoria, Australia; ^3^Department of Gastroenterology, The Royal Melbourne Hospital, Melbourne, Victoria, Australia; ^4^Department of Gastroenterology, Northern Health, Melbourne, Victoria, Australia; ^5^Department of Medical Education, University of Melbourne, Melbourne, Victoria, Australia

**Keywords:** digestive system endoscopies, diverticular diseases, gastrointestinal haemorrhage, melaena

## Abstract

Haemorrhage is one of the most common complications of jejunal diverticula, which is a challenge to diagnose as the anatomical location of the jejunum renders it inaccessible to standard upper endoscopy, while routine imaging modalities may miss subtle or intermittent bleeding. Male gender, increasing age and colonic diverticula are known risk factors for jejunal diverticula. Nonsteroidal anti-inflammatory drugs and corticosteroids increase gastrointestinal bleeding risk. We present a case of an 80-year-old male admitted to our hospital with syncope and melaena, in the setting of colonic diverticula, long-term aspirin and short-term corticosteroid therapy. Push enteroscopy, using a paediatric colonoscope, was pivotal to establishing the diagnosis of jejunal diverticular bleeding after gastroduodenoscopy and computed tomography (CT) angiogram were negative. Management was conservative with repeat push enteroscopy confirming the cessation of bleeding. Clinicians should consider this diagnosis when there are clinical signs of gastrointestinal bleeding in patients with known risk factors for jejunal diverticula and no evidence of location on gastroduodenoscopy, colonoscopy or imaging. We advise that push enteroscopy is performed early during the diagnostic workup to assist in identifying jejunal sources of bleeding and initiating management. However, as reflected by our case, jejunal diverticular haemorrhage may be amenable to conservative measures.

## 1. Introduction

Diverticular disease of the small bowel is rare, with an incidence of 0.01%–2.3% in clinical studies and up to 4.6% in autopsy studies [1]. They occur most commonly in the duodenum, followed by the jejunum and ileum [2]. Although most cases of small bowel diverticulosis are asymptomatic, they can be complicated by haemorrhage which is difficult to diagnose and potentially fatal [3, 4]. In this case report, we discuss a case of an 80-year-old male with clinically significant gastrointestinal bleeding secondary to a jejunal diverticulum, which was diagnostically challenging and ultimately managed conservatively. We highlight the diagnostic investigations performed and provide a review of the literature surrounding this condition.

## 2. Case Presentation

An 80-year-old male presented to our hospital following a syncopal episode and a three-day history of melaena. His past medical history was significant for ischaemic heart disease (IHD), chronic obstructive pulmonary disease (COPD), type 2 diabetes, hypertension, colonic diverticulosis and previous appendicectomy. He was taking 100 mg of aspirin daily for secondary prevention of IHD and had completed a weaning course of prednisolone (starting at 50 mg daily and dose reduced by 12.5 mg every three days) and a five-day course of antibiotics (amoxicillin 1 g 8 hourly and doxycycline 100 mg twice daily) for an infective exacerbation of COPD for which he was discharged from hospital nine days prior. His other medications included ascorbic acid 1000 mg daily, empagliflozin 25 mg daily, felodipine 10 mg daily, fluticasone (200 *μ*g)/umeclidinium (62.5 *μ*g)/vilanterol (25 *μ*g) inhaler once daily, gliclazide modified release 120 mg daily, glyceryl trinitrate sublingual spray 400 *μ*g as required, linagliptin 2.5 mg twice daily, metformin 1000 mg twice daily, pantoprazole 40 mg daily (while on prednisolone), ramipril 10 mg daily, salbutamol inhaler 100 *μ*g as required and simvastatin 40 mg daily.

During the initial assessment in the emergency department, he appeared alert and well with no sign of injury. His blood pressure was 136/60 mmHg, pulse was 100 beats/min, respiratory rate was 18 breaths/min and oxygen saturation was 97% on room air, and he was afebrile. The abdomen was soft and nontender. Digital per rectal examination showed melaena. The haemoglobin was 76 g/L from a baseline of 146 g/L nine days prior (normal = 130 g/L to 180 g/L), urea was 21.5 mmol/L (normal = 3 mmol/L to 10 mmol/L), creatinine was 86*μ*mol/L (normal = 60 *μ*mol/L to 110 *μ*mol/L) and lactate was 3 mmol/L (normal ≤ 2.3 mmol/L), and there was no coagulopathy. Glasgow-Blatchford Bleeding Score was 14 [5], AIMS65 was 2 [6] and Rockall Score (pre-endoscopy) was 5 [7].

He was treated as a suspected upper gastrointestinal bleed, receiving three units of packed red blood cells and an intravenous esomeprazole infusion administered at a rate of 8 mg per hour. Gastroduodenoscopy to the 2nd part of the duodenum performed thirty hours following presentation showed a small hiatus hernia, gastritis and multiple nonbleeding duodenal ulcers with no stigmata of recent bleeding. He remained haemodynamically stable but continued to experience melaena and a decreasing haemoglobin level on the ward, despite multiple blood transfusions. A *Helicobacter pylori* IgG serological test was conducted, which returned a positive result. However, due to a delay in receiving the result and the priority of managing ongoing bleeding, *Helicobacter pylori* antibiotic therapy was not initiated during the admission. The small shallow duodenal ulcers, which could have been *Helicobacter*-induced, were not considered the primary cause of the significant melaena and anaemia experienced by the patient. By day seven of admission, he had received a total of 10 units of packed red blood cells and an iron infusion. An abdominal CT angiogram was conducted to localise the source of bleeding and plan the appropriate therapeutic procedure. This approach aimed to minimise unnecessary procedures in a patient with significant comorbidities. The CT angiogram showed extensive diverticular disease involving the sigmoid and descending colon, with no evidence of diverticulitis or acute gastrointestinal haemorrhage.

**Figure 1:**
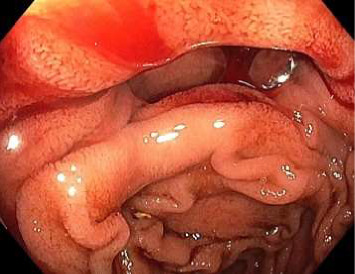
Bleeding culprit jejunal diverticulum.

**Figure 2:**
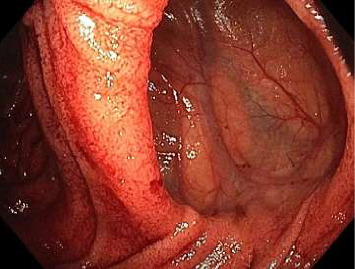
Nonbleeding, nonculprit jejunal diverticulum.

**Figure 3:**
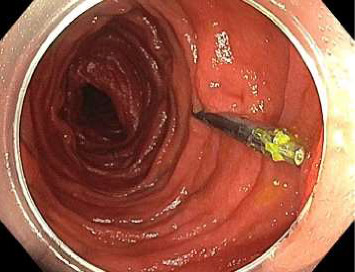
Previously placed clip.

**Figure 4:**
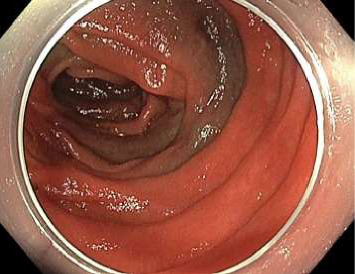
Previously placed tattoo.

A combined push enteroscopy and subsequent colonoscopy were planned on day ten of admission to further evaluate the gastrointestinal tract. During push enteroscopy using a paediatric colonoscope (Olympus PCF-H190TL), a diffusely bleeding culprit large jejunal diverticulum was identified approximately 140 cm distal to the incisors, 50 cm distal to the pylorus (Figure 1). The exact source of bleeding within the diverticulum was unclear due to fresh and clotted blood rapidly obscuring views. Furthermore, the paediatric colonoscope could only reach 5 cm proximal to the opening of the diverticulum before gastric looping prevented further distal movement. Several other medium-sized nonbleeding, nonculprit jejunal diverticula were also found proximally (Figure 2). A haemostatic clip (Cook, Instinct Plus™ Endoscopic Clipping Device) was placed 5 cm proximal to the bleeding diverticulum and a submucosal ink tattoo was also placed 10 cm proximal to it, to mark the location for interventional radiology and/or surgery (Figures 3 and 4). During the procedure, the patient developed haemodynamic instability requiring metaraminol. Given the sudden deterioration and unclear bleeding site, colonoscopy was abandoned and a repeat abdominal CT angiogram was performed with the aim of guiding embolisation with interventional radiology. A general surgeon was also consulted for consideration of surgical management in the case of failed embolisation or further deterioration. Interestingly, no active gastrointestinal haemorrhage was observed on repeat CT angiogram, and following resuscitation, the patient maintained haemodynamic stability.

**Figure 5:**
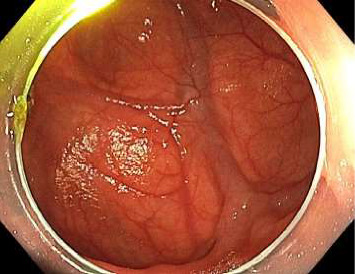
Large diverticulum just distal (anal side) to the clip, no active bleeding.

Push enteroscopy was repeated the following day and a large jejunal diverticulum 5 cm distal to the haemostatic clip was identified with no further stigmata of active bleeding (Figure 5). Unlike the first push enteroscopy, during this second procedure, the endoscope was able to enter the culprit diverticulum and inspect the entire internal mucosa. The decision was made to withhold the patient's aspirin and to continue monitoring as an inpatient. A technetium-99m (Tc-99)–labelled red blood cell scan was performed on day fourteen of admission due to its high sensitivity in detecting gastrointestinal bleeding; no active bleeding was observed. From day fourteen onwards, the patient stopped experiencing melaena and his haemoglobin began incrementing in the absence of blood transfusions. He was discharged on day seventeen of admission with a follow-up clinic visit planned in two to three weeks, which was the earliest feasible time to consider recommencing aspirin. However, due to logistical issues in scheduling the outpatient clinic, exacerbated by the COVID-19 pandemic, and difficulties in contacting the patient, aspirin was not restarted until two months later. At a clinic visit four months after restarting aspirin, the patient showed no signs of rebleeding, as indicated by stable haemoglobin levels and the absence of melaena, per rectal bleeding, weight loss or abdominal pain.

Images from initial push enteroscopy (day ten of admission) are given in Figures 1 and 2. Images from repeat push enteroscopy (day eleven of admission) are given in Figures 3, 4 and 5.

## 3. Discussion

Diverticula are outpouchings of the tubular structure of the gastrointestinal tract. True diverticula comprise all three layers of the wall and false diverticula comprise only the mucosal or submucosal layers [8]. Small bowel diverticula can be congenital or acquired [8]. Meckel's diverticulum is the most common congenital small bowel diverticulum, occurring in 2%-3% of the population [9]. Small bowel pseudo-diverticula are rarely seen clinically; however, they have a reported prevalence of up to 7% in the general population based on studies using autopsy and radiology data [10]. They develop due to abnormalities in peristalsis, intestinal dyskinesis and high intraluminal pressure [11]. These abnormalities cause herniation of the mucosa, submucosa and serosa through the muscular layer of the bowel where the vasa recta enters the muscularis propria [11]. Most small bowel diverticula are duodenal, reflecting 79% of cases [12]. Regarding diverticulosis of the jejunoileal segment, 80% occur in the jejunum, 15% in the ileum and 5% are in both [3]. Jejunal diverticula are usually multiple in number and mostly found in the proximal jejunum, on the mesenteric border [13]. They have a predominance in males, the elderly and those with colonic diverticula, risk factors largely reflective of our patient [14, 15].

Although they are usually asymptomatic, they can manifest with vague abdominal symptoms such as bloating, distention and pain, mimicking other gastrointestinal conditions which contributes to delayed or missed diagnosis [16]. Severe complications include obstruction, fistulas, diverticulitis, abscess formation, volvulus, intussusception, diverticular perforation and haemorrhage, which develop in approximately 10%–30% of patients [3, 15]. Haemorrhage is one of the most common complications of jejunal diverticula and occurs in 3.4%–8.1% of cases [3, 16]. Bleeding has been attributed to mucosal ulceration which compromises blood vessels, the damaging effects of digestive fluids released from ectopic pancreatic tissue or gastric mucosa within diverticula and administration of nonsteroidal anti-inflammatory drugs and antiplatelet or anticoagulant agents [11, 14, 17, 18]. Corticosteroids are known to increase the risk of gastrointestinal bleeding; however, we did not identify any case reports linking their use to jejunal diverticular bleeding [19]. In a case series on jejunoileal haemorrhage, associated presentations in order of decreasing frequency included tarry stool, bloody stool, shock and coffee ground vomitus [2].

When gastrointestinal bleeding is suspected, upper and lower endoscopies are strongly indicated following appropriate resuscitation [15, 20]. Direct visualisation of the mucosa through endoscopy allows for identification of bleeding, as well as the provision of haemostatic therapy and tissue sampling for suspected malignancies [20]. If a bleeding source is not identified on endoscopy, CT angiography is recommended in the acute setting, followed by invasive catheter-assisted mesenteric angiography [11, 14, 15, 20]. CT angiography can detect acute active intestinal bleeding with a sensitivity of 85.2% and specificity of 92.1% [21]. Mesenteric angiography can detect bleeding rates as low as 0.1–0.5 mL/min [18]. The disadvantages of angiography include risk of ischaemia, high radiation dose, need for contrast (an issue in renal impairment) and false-negative results, which can occur if the patient is not actively bleeding during imaging [4, 20]. In the presence of renal impairment or more subtle bleeding, technetium-99m–labelled red blood cell scintigraphy can also be performed [20, 22]. It detects bleeding rates as low as 0.2 mL/min with a sensitivity of 93% and specificity of 95%; it has a false localisation rate of 22% [3].

Transcatheter embolisation has been successful in treating bleeding jejunal diverticula in previous case reports, albeit carrying a risk of ischaemia [16]. Embolisation should occur rapidly following a positive angiogram to increase the likelihood of identifying and treating active bleeding [20]. In the haemodynamically unstable patient, surgical intervention (suture and ligation of the bleeding vessel and/or resection of the disease segment of the jejunum) is the definitive treatment [3, 14]. Surgery yields a higher diagnostic accuracy for jejunoileal diverticular haemorrhage compared to enteroscopy and CT imaging modalities [2]. Surgery has also shown a lower rebleeding rate than interventional radiology or enteroscopic treatment but is associated with longer hospital stays and greater blood transfusion volumes [2].

Advances in enteroscopy have improved the diagnosis and treatment of jejunal diverticular haemorrhage, reducing the need for surgery, by allowing for greater access and visualisation of the small bowel [23]. Enteroscopy can be categorised into push enteroscopy and device-assisted deep enteroscopy (single- and double-balloon enteroscopy). Double-balloon enteroscopy can evaluate the entire small bowel; however, it requires highly specialised skills and equipment that are not accessible in many health services [23]. Push enteroscopy is more widely available across hospitals and allows visualisation of the proximal small bowel to 50–100 cm beyond the ligament of Treitz [20]. Although dedicated push enteroscopes (length of 160–250 cm) are commercially available, paediatric colonoscopes are often used for push enteroscopy due to their widespread accessibility, good shaft flexibility and small insertion tube diameter [20]. Haemostatic techniques which have been used to successfully treat jejunal diverticular bleeding during enteroscopy include clipping, argon plasma coagulation, endoscopic band ligation and epinephrine injection [15, 24]. The most ideal haemostatic therapy remains unknown and is largely dependent on endoscopists' preference and experience [20].

As described in our case, achieving haemostasis endoscopically is not always possible during active bleeding. Views can become obscured by blood, making it difficult to identify and treat the exact source of bleeding [23]. The thin walls of the small intestine are also vulnerable to perforation during haemostatic attempts [25]. In these circumstances, marking the location with a tattoo and/or a clip can assist in identifying the bleeding source during subsequent diagnostic procedures, such as angiography, surgery or repeat enteroscopy. Location marking was essential to locate the culprit jejunal diverticulum on repeat push enteroscopy.

Here we present a case of clinically significant jejunal diverticular bleeding in the setting of aspirin and corticosteroid therapy, managed conservatively. Push enteroscopy, using a paediatric colonoscope, was pivotal to establishing the diagnosis and, when repeated, in confirming that bleeding had stopped following identification of the previously bleeding jejunal diverticulum.

## 4. Conclusion

Jejunal diverticular haemorrhage is challenging to diagnose, particularly when bleeding is intermittent. Clinicians should consider this diagnosis when there are clinical signs of gastrointestinal bleeding in patients with known risk factors and no evidence of location on gastroduodenoscopy, colonoscopy or imaging. Thus, we advise that push enteroscopy is performed early in the diagnostic workup to assist in identifying jejunal sources of bleeding and initiating treatment where able. Our case report demonstrates that jejunal diverticular haemorrhage can be managed conservatively through supportive measures and cessation of precipitating agents.

## Data Availability

The demographic, biochemical, haemodynamic and endoscopic data used to support the findings of this study are included within the article.
